# Serum soluble lectin‐like oxidized low‐density lipoprotein receptor‐1 as a biomarker of delayed cerebral ischemia after aneurysmal subarachnoid hemorrhage

**DOI:** 10.1002/brb3.1517

**Published:** 2020-01-14

**Authors:** Qun Lin, Hua‐Jun Ba, Jun‐Xia Dai, Jun Sun, Chuan Lu, Mao‐Hua Chen, Xian‐Dong Chen, Jian‐Yong Cai

**Affiliations:** ^1^ Department of Neurosurgery The Central Hospital of Wenzhou City Wenzhou China

**Keywords:** aneurysmal subarachnoid hemorrhage, delayed cerebral ischemia, lectin‐like oxidized low‐density lipoprotein receptor‐1, severity

## Abstract

**Objective:**

Delayed cerebral ischemia (DCI) greatly contributes to the high morbidity and mortality of aneurysmal subarachnoid hemorrhage (aSAH) patients. Expression of lectin‐like oxidized low‐density lipoprotein receptor‐1 (LOX‐1) was substantially raised in the basilar arterial wall of SAH rabbits. We attempted to ascertain the relationship between serum soluble LOX‐1 (sLOX‐1) levels and the occurrence of DCI after aSAH.

**Materials and methods:**

We enrolled 125 aSAH patients and 125 healthy controls. Serum sLOX‐1 levels were quantified using commercial enzyme‐linked immunosorbent assay kit. The relationship between sLOX‐1 levels and DCI was analyzed utilizing the multivariate logistic regression analysis.

**Results:**

Serum sLOX‐1 levels were significantly higher in stroke patients than in controls (median: 1,450.2 vs. 445.7 pg/ml, *p* < .001). Serum sLOX‐1 levels were highly correlated with World Federation of Neurological Surgeons (WFNS) scores, Hunt‐Hess scores, and modified Fisher scores (*r* = .574, .625, and .569, respectively). Forty‐two patients (33.6%) experienced DCI. Serum sLOX‐1 > 1,450.2 pg/ml, WFNS scores and modified Fisher scores were the independent predictors of DCI. Under receiver operating characteristic curve, serum sLOX‐1 levels exhibited a significant discriminatory capability (area under curve 0.825, 95% confidence interval 0.747–0.887). The predictive power of serum sLOX‐1 levels was similar to those of WFNS scores and modified Fisher grade (both *p* > .05). Moreover, serum sLOX‐1 levels significantly improved their predictive capability (both *p* < .05).

**Conclusions:**

Serum soluble LOX‐1, in positive association with hemorrhagic severity, appears to have the potential to become a promising predictor of DCI after aSAH.

## Graphical abstract

There is a substantial elevation in serum sLOX‐1 levels after aSAH. There is an intimate correlation of serum sLOX‐1 levels with hemorrhagic severity. There is an independent association of serum sLOX‐1 levels with delayed cerebral ischemia.

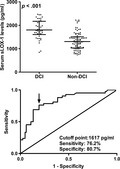

## 1 INTRODUCTION

Aneurysmal subarachnoid hemorrhage (aSAH) is one of the commonest cerebrovascular diseases and is characterized by a high mortality (Etminan & Macdonald, 2017; Grimm, 2015; Serrone, Maekawa, Tjahjadi, & Hernesniemi, 2015). After aSAH, cerebral vasospasm can induce the decreased cerebral blood flow, subsequently resulting in delayed cerebral ischemia (DCI), and finally leading to severe neurological deficit and even death (Burrell et al., 2016; Francoeur & Mayer, 2016; Geraghty & Testai, 2017). The accumulating evidence shows microvasculature, coagulation and fibrinolytic systems, cortical spreading depressions, and the immune system might participate in pathophysiological process of DCI (Bacigaluppi et al., 2015; Carr, Zuckerman, & Mocco, 2013; Foreman, 2016). However, the actual pathogenesis of DCI still warrants to be explored.

Oxidized low‐density lipoprotein (ox‐LDL) and its receptor, lectin‐like ox‐LDL receptor‐1 (LOX‐1), is involved in the pathogenesis of atherosclerosis by mediating various reactions and processes (Aoyama, Fujiwara, Masaki, & Sawamura, 1999; Li & Mehta, 2000; Mehta, Chen, Hermonat, Romeo, & Novelli, 2006; Sawamura et al., 1997). Ox‐LDL, via binding to LOX‐1, increases intracellular reactive oxygen species, leads to a reduction in nitric oxide availability and down‐regulates endothelial nitric oxide synthase and initiates the inhibition of vasodilatation (Ma et al., (2006); Sakurai & Sawamura, 2003). Ox‐LDL and LOX‐1 were increased in the basilar arterial wall of rabbits with SAH (Matsuda et al., 2014). So, it is assumed that LOX‐1 might be implicated the pathogenesis of DCI. Soluble LOX‐1 (sLOX‐1) is generated through proteolytic cleavage of the extracellular domain of LOX‐1 and, therefore, sLOX‐1 can be utilized to distinguish acute coronary syndrome, acute spontaneous intracerebral hemorrhage, and acute ischemic stroke (Inoue et al., 2019; Misaka et al., 2014; Yokota et al., 2016). However, sLOX‐1 in the peripheral blood has not been explored in patients with aSAH. In this study, we intended to determine whether there is a difference in sLOX‐1 levels between patients who incurred an aSAH and those who did not, and between patients who suffered from a DCI and those who did not. We also investigated the usefulness of circulating sLOX‐1 as an early predictor of DCI after aSAH.

## 2 MATERIALS AND METHODS

### 2.1 Study population

In this prospective observational study, we consecutively enrolled first‐ever nontraumatic SAH patients who were admitted to the Department of Neurosurgery at our hospital within 24 hr of stroke onset from March 2015 to May 2019, and fulfilled the selection criteria as follows: (a) SAH caused by a single intracranial aneurysm confirmed via computerized tomography angiography or digital subtraction angiography, and (b) clipping or interventional treatment of the aneurysm was carried on within 48 hr after admission. We also removed those patients with (a) any type of surgery or acute or chronic infection within recent a month; (b) previous other neurological diseases, for example, ischemic stroke, hemorrhagic stroke, and severe head trauma; (c) prior use of antiplatelet medication, anticoagulant drugs, or immunosuppressants; and (d) other systemic diseases such as autoimmune disease, uremia, cirrhosis, cancer, chronic lung diseases, and chronic heart diseases. Additionally, a group of control individuals, free of other diseases, were recruited. This study was performed in accordance with the Code of Ethics of the World Medical Association (Declaration of Helsinki) and was also based on the ethical standards of our institution. This study was approved by the institutional ethics committees at our institution and the written informed consent was obtained from participants or their relatives.

### 2.2 Patient management and clinical assessment

Treatment for aSAH was performed according to the guidelines from the American Heart Association and American Stroke Association (Connolly et al., 2012). Critical care management was based on the guidelines from the Neurocritical Care Society (Diringer et al., 2011). Hypertensive hypervolemic therapy was only done for patients with symptomatic vasospasm. We set target systolic blood pressure to 180–220 mmHg for poor‐grade patients with severe angiographic vasospasm (Komotar et al., 2009). The modified Fisher grading scale was utilized to estimate the amount of subarachnoid blood presented on the head computerized tomography scan at admission. Clinical severity of aSAH was assessed on the basis of the initial Hunt and Hess scale, and World Federation of Neurosurgical Societies (WFNS) grade. In this study, admission WFNS grade, modified Fisher grade or Hunt‐Hess grade ≥3, was identified as severe aSAH.We collected basic information (such as age and gender), aneurysmal morphological characteristics including shape, size and position, and other radiological parameters (such as acute hydrocephalus and intraventricular hemorrhage). Brain computerized tomography scans were carried out at days 1, 7, 14, and 30 following surgery. DCI was considered according to the criteria below: (a) clinical deterioration (namely, a new focal deficit, decrease in level of consciousness, or both), and/or (b) a new infarct on head computerized tomography scan that was invisible at admission or immediately postoperatively and cannot be attributed to other causes by means of clinical assessment, imaging of the brain, and appropriate laboratory studies (Frontera et al., 2009).

### 2.3 Measurements

Venous blood was drawn for patients at admission and for controls at study entrance. Blood samples were collected in sterile tubes, next centrifuged at 3,000 *g* for 15 min, aliquoted into 1.5‐ml tubes and preserved at −80°C until analysis. sLOX‐1 was detected with enzyme immunoassay, commercially available from NK Medico Co. Ltd. Quantifications were completed in duplicate, and the results were averaged. Samples with obvious hemolysis were not utilized for further determinations. All measurements were completed in batches every 3 months by the same technician who was completely inaccessible to the clinical information.

### 2.4 Statistical analysis

SPSS 19.0 for windows (SPSS Inc.), Stata/SE 12.0 (StataCorp) and MedCalc 9.6.4.0 (MedCalc Software) were performed for statistical analysis. Kolmogorov–Smirnov test was carried out to investigate data distribution. Because all continuous variables were not normally distributed, they were reported as median (interquartile range) and analyzed using the Mann–Whitney *U* test. Categorical variables were presented as counts (percentage) and analyzed using the Pearson chi‐square test or Fisher exact test as appropriate. Bivariate correlations were analyzed by the Spearman's rank correlation test. Predictors of DCI were analyzed using the binary logistic regression analysis. All variables with *p* values < .05 from univariate analyses were incorporated in multivariate analysis. Area under the curve (AUC) was derived from the receiver operating characteristic (ROC) curve. The corresponding sensitivity, specificity, and Youden index were also calculated using the best threshold for serum sLOX‐1 levels. A combined binary logistic regression model was configured to verify additive effect of serum sLOX‐1 to other variables. Using Stata/SE 12.0 statistical software, the calculated least patient number was 102. A two‐sided *p* value < .05 was considered statistically significant.

## 3 RESULTS

### 3.1 Subjects

All aSAH patients were screened during the enrollment period and eventually a total of 125 patients were analyzed. Simultaneously, 125 controls, with similar age and gender percentage, were chosen. Among those aSAH patients including 74 females and 51 males, age ranged from 21 to 77 years (median, 51 years; interquartile range, 38–62 years). The median values of admission WFNS scores, modified Fisher scores and Hunt‐Hess scores were 3 (range, 1–5; interquartile range, 1–4), 2 (range, 1–4; interquartile range, 2–3), and 2 (range, 1–5; interquartile range, 2–3), respectively. Target aneurysms of 100 patients were located in anterior circulation and other remainders, in posterior circulation. Cystic aneurysms were found in 106 patients and noncystic ones were revealed in other 19 cases. Median diameter of target aneurysm was 8.0 mm (range, 2.0–20.0 mm; interquartile range, 5.0–12.0 mm). As regards aneurysmal treatment, a total of 62 patients underwent a surgical clipping and 63 patients, an endovascular coiling. Acute hydrocephalus occurred among 20 patients, 42 patients suffered from DCI and 18 patients experienced intraventricular hemorrhage. Finally, an external ventricular drainage was done among 28 patients. Patients were admitted from 1.0 to 24.0 hr after stroke (median, 11.5 hr; interquartile range, 6.6–14.7 hr). Their blood samples were collected from 3.9 to 26.0 hr following aSAH (median, 13.9 hr; interquartile range, 10.8–19.7 hr). Via laboratory test, there was 12.8 mmol/L at median value of blood glucose levels (range, 5.9–24.3 mmol/L; interquartile range, 11.3–16.8 mmol/L). Noninvasive blood pressure measurement showed that the median values of systolic arterial pressure, diastolic arterial pressure, and mean arterial pressure were 147 mmHg (range, 101–187 mmHg; interquartile range, 138–164 mmHg), 87 mmHg (range, 53–109 mmHg; interquartile range, 71–94 mmHg), and 106 mmHg (range, 74–129 mmHg; interquartile range, 96–115 mmHg), respectively.

### 3.2 Serum sLOX‐1 levels

**Figure 1:**
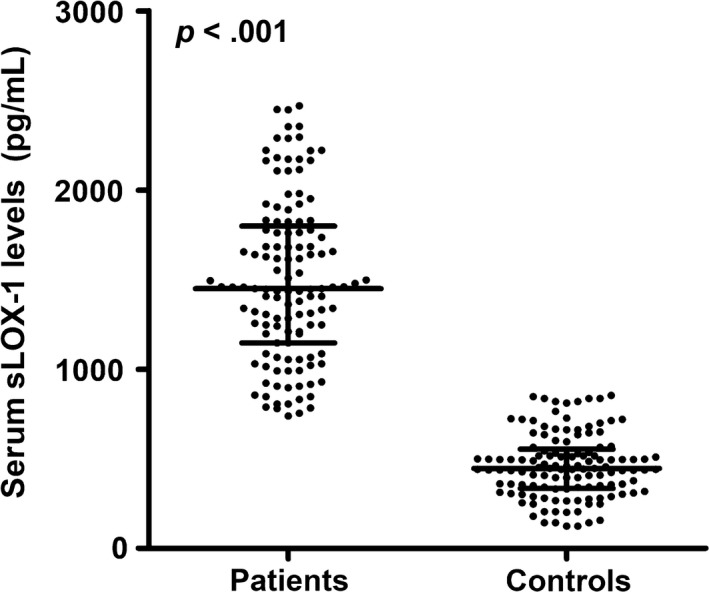
Difference of serum soluble lectin‐like oxidized low‐density lipoprotein receptor‐1 (sLOX‐1) levels between healthy controls and patients with aneurysmal subarachnoid hemorrhage

**Figure 2:**
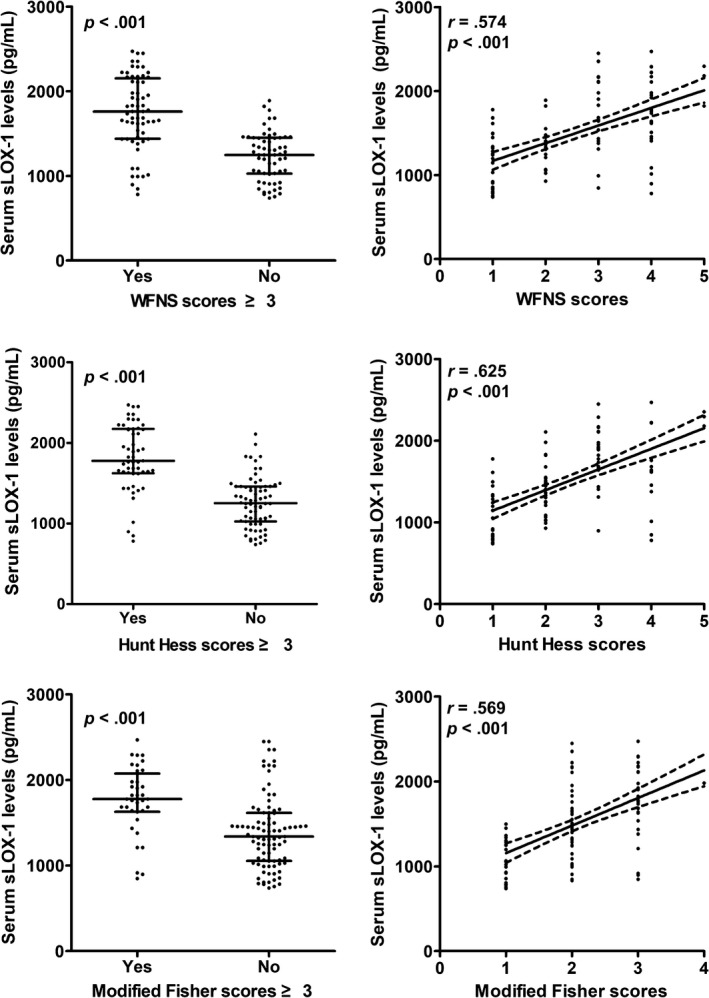
Relationship between serum soluble lectin‐like oxidized low‐density lipoprotein receptor‐1 (sLOX‐1) levels and World Federation of Neurological Surgeons (WFNS) scores, modified Fisher scores, and Hunt‐Hess scores in patients with aneurysmal subarachnoid hemorrhage

Figure 1 shows that serum sLOX‐1 levels were actually significantly higher in patients than in controls. In Figure 2, serum sLOX‐1 levels were highly correlated with admission WFNS, modified Fisher, and Hunt‐Hess scores. In addition, patients with severe aSAH (WFNS scores, modified Fisher scores, or Hunt‐Hess scores ≥ 3) exhibited significantly higher serum sLOX‐1 levels than other remainders (Figure 2).

### 3.3 Serum sLOX‐1 levels and DCI

**Figure 3:**
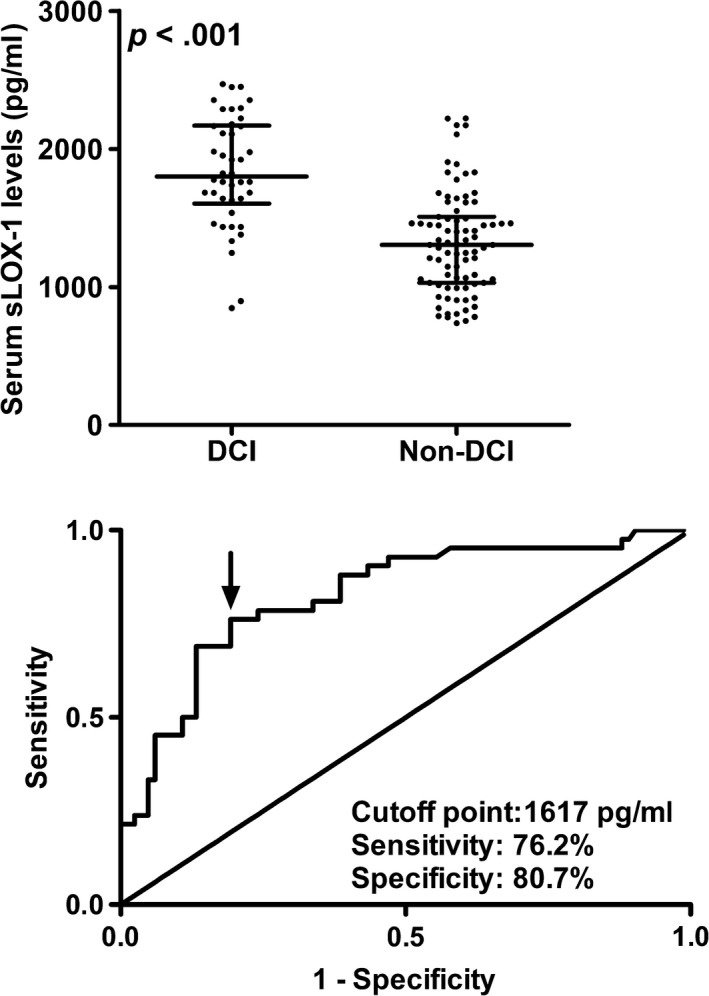
Predictive significance of serum soluble lectin‐like oxidized low‐density lipoprotein receptor‐1 (sLOX‐1) levels for delayed cerebral ischemia (DCI)

In Figure 3, serum sLOX‐1 levels were substantially elevated in patients who presented with DCI, as compared with those who did not. Furthermore, serum sLOX‐1 levels were bifurcated based its median value (1,450.2 pg/ml). It was revealed that serum sLOX‐1 levels >1,450.2 pg/ml and other variables listed in Table 1, such as WFNS scores, modified Fisher scores, Hunt‐Hess scores, external ventricular drainage, acute hydrocephalus, intraventricular hemorrhage, and blood glucose levels, were strongly associated with occurrence of DCI. Table 2 displayed their odds ratio and 95% confidence interval values. Moreover, we incorporated the preceding significant variables in the binary logistic regression model and thereby found that WFNS scores modified Fisher scores and serum sLOX‐1 levels >1,450.2 pg/ml emerged as the independent predictors for occurrence of DCI (Table 3).

**Table 1:** Comparison of demographic and clinical data in patients with aneurysmal subarachnoid hemorrhage according to the occurrence of delayed cerebral ischemia

	Delayed cerebral ischemia		*p* value
	Presence	Absence	
Gender (female/male)	24/18	50/33	.739
Age (years)	48 (38–65)	54 (39–62)	.527
WFNS scores	4 (3–4)	2 (1–3)	<.001
Modified Fisher scores	3 (2–3)	2 (1–2)	<.001
Hunt‐Hess scores	3 (3–4)	2 (1–2)	<.001
Aneurysm in anterior circulation	32 (76.2%)	68 (81.9%)	.449
Cystic aneurysm	34 (81.0%)	72 (86.8%)	.394
Aneurysmal diameter (mm)	9.0 (4.0–12.0)	7.6 (5.6–12.0)	.453
Clipping treatment of aneurysm	20 (47.6%)	42 (50.6%)	.753
External ventricular drainage	16 (38.1%)	12 (14.5%)	.003
Acute hydrocephalus	12 (28.6%)	8 (9.6%)	.006
Intraventricular hemorrhage	12 (28.6%)	6 (7.2%)	.001
Admission time (hr)	10.1 (6.3–13.0)	12.7 (7.2–15.4)	.068
Blood‐collection time (hr)	14.6 (11.1–20.2)	13.9 (10.5–19.5)	.792
Serum sLOX−1 levels > 1,450.2 pg/ml	34 (81.0%)	28 (33.7%)	<.001
Blood glucose level (mmol/L)	15.6 (11.3–20.5)	12.6 (11.3–15.6)	.024
Systolic arterial pressure (mmHg)	148 (144–164)	146 (132–165)	.224
Diastolic arterial pressure (mmHg)	90 (77–95)	86 (67–92)	.092
Mean arterial pressure (mmHg)	109 (97–116)	106 (94–113)	.112

Note

Continuous variables were reported as median (interquartile range) and analyzed using the Mann–Whitney U test. Categorical variables were presented as counts (percentage) and compared using the Pearson chi‐square test or Fisher exact test. WFNS indicates World Federation of Neurological Surgeons; sLOX‐1 denotes soluble lectin‐like oxidized low‐density lipoprotein receptor‐1.

**Table 2:** Factors associated with the occurrence of delayed cerebral ischemia using univariate logistic regression analysis in patients with aneurysmal subarachnoid hemorrhage

	Odds ratio	95% confidence interval	*p* value
Gender (female/male)	0.880	0.415–1.868	.739
Age (years)	0.991	0.966–1.017	.510
WFNS scores	4.873	2.805–8.466	<.001
Modified Fisher scores	13.452	5.514–32.816	<.001
Hunt‐Hess scores	5.714	3.100–10.531	<.001
Aneurysm in anterior circulation	1.417	0.574–3.498	.450
Cystic aneurysm	0.649	0.239–1.761	.396
Aneurysm diameter (mm)	0.965	0.879–1.058	.447
Clipping treatment of aneurysm	1.127	0.536–2.369	.753
External ventricular drainage	3.641	1.521–8.717	.004
Acute hydrocephalus	3.750	1.394–10.089	.009
Intraventricular hemorrhage	5.133	1.766–14.919	.003
Admission time (hr)	0.950	0.886–1.019	.155
Blood‐collection time (hr)	1.002	0.935–1.073	.965
Serum sLOX‐1 levels > 1,450.2 pg/ml	8.348	3.413–20.422	<.001
Blood glucose level (mmol/L)	1.151	1.049–1.263	.003
Systolic arterial pressure (mmHg)	1.012	0.993–1.032	.216
Diastolic arterial pressure (mmHg)	1.022	0.993–1.051	.137
Mean arterial pressure (mmHg)	1.024	0.995–1.054	.109

Note

WFNS indicates World Federation of Neurological Surgeons; sLOX‐1 denotes soluble lectin‐like oxidized low‐density lipoprotein receptor‐1.

**Table 3:** Parameters related to the occurrence of delayed cerebral ischemia using multivariate logistic regression analysis in patients with aneurysmal subarachnoid hemorrhage

	Odds ratio	95% confidence interval	*p* value
WFNS scores	5.271	1.577–17.615	.007
Modified Fisher scores	11.002	2.545–47.560	.001
Serum sLOX‐1 levels > 1,450.2 pg/ml	5.639	1.471–21.617	.012

Note

WFNS indicates World Federation of Neurological Surgeons; sLOX‐1 denotes soluble lectin‐like oxidized low‐density lipoprotein receptor‐1.

Figure 3 shows that an optimal value of serum sLOX‐1 levels (1617 pg/ml) was selected, which yielded the corresponding sensitivity and specificity values (Youden index = 0.569). Moreover, its predictive ability (AUC 0.825, 95% confidence interval (CI) 0.747–0.887) was equivalent to those of WFNS scores (AUC 0.867, 95% CI 0.795–0.921, *p* = .310) and modified Fisher scores (AUC 0.849, 95% CI 0.773–0.906, *p* = .589). Using a combined binary logistic regression model, serum sLOX‐1 levels significantly improved AUCs of WFNS scores and modified Fisher scores to 0.903 (95% CI 0.838–0.949, *p* = .035) and 0.898 (95% CI 0.831–0.945, *p* = .028), respectively.

## 4 DISCUSSION

Delayed cerebral ischemia, a severe complication, occurs very often within 2 weeks of hemorrhagic stroke. DCI can cause brain injury and result in neurological defect and even death of aSAH patients (Budohoski et al., 2012; Duan et al., 2018). Hence, its early identification might be beneficial for optimizing care and improving allocation of healthcare resources among aSAH patients. WFNS scores, modified Fisher score, and Hunt‐Hess score are highly associated with hemorrhagic severity after aSAH and are usually recorded to predict occurrence of DCI. However, DCI is a complex process with underlying complicated pathophysiological mechanisms (Bacigaluppi et al., 2015; Carr et al., 2013; Foreman, 2016). As a consequence, some biomarkers have been fully investigated for their predictive ability for DCI in recent decades (Yang et al., 2019; Zhu et al., 2019).

LOX‐1 emerged as a scavenger receptor present primarily on vascular endothelial cells. LOX‐1 can combine with ox‐LDL in endothelial cells and play a key role in vascular dysfunction, including apoptosis and inhibition of vasodilatation (Ma et al., (2006); Sakurai & Sawamura, 2003). LOX‐1 is also expressed in the intracerebral artery and its expression can be up‐regulated significantly in rabbits with SAH (Matsuda et al., 2014). In a previous case report, remarkable expressions of LOX‐1 was found in hypertrophic media outside the intima in ruptured dissection using immunohistochemical examination (Saito, Fujimura, Inoue, Shimizu, & Tominaga, 2010). Moreover, among patients with acute intracerebral hemorrhage or acute ischemic stroke, circulating sLOX‐1 levels were substantially raised, as compared to healthy controls (Inoue et al., 2019; Yokota et al., 2016). Our clinical investigation showed that serum sLOX‐1 levels were markedly raised after aSAH. Overall, LOX expression might be linked to human cerebral vascular dysfunction after aSAH.

Generally, WFNS scores, modified Fisher scores, and Hunt‐Hess scores are widely used to assess hemorrhagic severity after aSAH. Commonly, severe aSAH can be referred to as WFNS scores, modified Fisher scores, or Hunt‐Hess scores ≥3 (Drake, 1988; Frontera et al., 2006; Mericle et al., 2006). In the current study, we confirmed that increasing circulating sLOX‐1 levels were intimately correlated with rising disease severity indicated by the above‐mentioned severity scales, namely, WFNS scores, modified Fisher scores, and Hunt‐Hess scores. Meanwhile, patients with severe aSAH exhibited higher serum sLOX‐1 levels, as compared to other remaining ones. Taken together, sLOX‐1 in serum might be measured to assess hemorrhagic severity among aSAH patients.

In order to determine whether serum sLOX‐1 was an independent predictor for occurrence of DCI, we at first used univariate analysis to discern the factors highly associated with occurrence of DCI. We thereby found that serum sLOX‐1 levels and other variables, including WFNS scores, Hunt‐Hess scores, and modified Fisher scores, were related to development of DCI. Then, such significant variables were incorporated in the multivariate logistic regression model. Interestingly, serum sLOX‐1 levels, WFNS scores, and modified Fisher scores retained as the independent predictors for development of DCI. Furthermore, in order to investigate the discriminatory capability for patients at risk of DCI, ROC curve was configured. We actually found that serum sLOX‐1 levels were able to predict DCI significantly. Moreover, its predictive ability was similar to those of WNFS scores and modified Fisher scores. More intriguingly, serum sLOX‐1 levels could be able to significantly improve the predictive capability of WFNS scores and modified Fisher score. In summary, serum sLOX‐1 might serve as a potential biomarker for predicting occurrence of DCI after aSAH.

In this study, some considerations should be mentioned. Laboratory test for serum sLOX‐1 levels is convenient because residual blood from standard blood draws on admission can be used for its determination, and moreover most hospitals have already been equipped to perform such analysis. However, its daily measurement is a little difficult, and moreover, sLOX‐1 expression might rise and fall over the 14‐day DCI window, so its clinical use warrants to be considered cautiously. In future, it might be helpful to track the change of serum sLOX‐1 levels over time.

## 5 CONCLUSIONS

To the best of my knowledge, it is the first study for measuring serum sLOX‐1 levels in a group of aSAH patients and further investigating its predictive ability for DCI. Of note, an elevation of serum sLOX‐1 levels is found after aSAH; serum sLOX‐1 levels are highly correlated with WNFS scores, modified Fisher scores, and Hunt‐Hess scores; serum sLOX‐1 emerges as an independent predictor for DCI; serum sLOX‐1 levels have similar discriminatory ability for DCI, as compared to WFNS scores and modified Fisher scores; and serum sLOX‐1 levels substantially improve the predictive values of WFNS scores and modified Fisher scores. Such data indicate that serum sLOX‐1 might have the potential to represent a promising biomarker for assessing stroke severity and predicting DCI in patients with aSAH.

## CONFLICT OF INTEREST

None declared.

## Data Availability

The data that support the findings of this study are available on request from the corresponding author. The data are not publicly available due to privacy or ethical restrictions.

## References

[brb31517-bib-0001] Aoyama, T. , Fujiwara, H. , Masaki, T. , & Sawamura, T. (1999). Induction of lectin‐like oxidized LDL receptor by oxidized LDL and lysophosphatidylcholine in cultured endothelial cells. Journal of Molecular and Cellular Cardiology, 31, 2101–2114. 10.1006/jmcc.1999.1041 10640439

[brb31517-bib-0002] Bacigaluppi, S. , Zona, G. , Secci, F. , Spena, G. , Mavilio, N. , Brusa, G. , … Fontanella, M. . (2015). Diagnosis of cerebral vasospasm and risk of delayed cerebral ischemia related to aneurysmal subarachnoid haemorrhage: An overview of available tools. Neurosurgical Review, 38, 603–618. 10.1007/s10143-015-0617-3 25732522

[brb31517-bib-0003] Budohoski, K. P. , Czosnyka, M. , Smielewski, P. , Kasprowicz, M. , Helmy, A. , Bulters, D. , … Kirkpatrick, P. J. . (2012). Impairment of cerebral autoregulation predicts delayed cerebral ischemia after subarachnoid hemorrhage: A prospective observational study. Stroke, 43, 3230–3237. 10.1161/STROKEAHA.112.669788 23150652

[brb31517-bib-0004] Burrell, C. , Avalon, N. E. , Siegel, J. , Pizzi, M. , Dutta, T. , Charlesworth, M. C. , & Freeman, W. D. (2016). Precision medicine of aneurysmal subarachnoid hemorrhage, vasospasm and delayed cerebral ischemia. Expert Review of Neurotherapeutics, 16, 1251–1262. 10.1080/14737175.2016.1203257 27314601 PMC5573230

[brb31517-bib-0005] Carr, K. R. , Zuckerman, S. L. , & Mocco, J. (2013). Inflammation, cerebral vasospasm, and evolving theories of delayed cerebral ischemia. Neurology Research International, 2013, 506584 10.1155/2013/506584 24058736 PMC3766617

[brb31517-bib-0006] Connolly, E. S. Jr , Rabinstein, A. A. , Carhuapoma, J. R. , Derdeyn, C. P. , Dion, J. , Higashida, R. T. , … Vespa, P. . (2012). Guidelines for the management of aneurysmal subarachnoid hemorrhage: A guideline for healthcare professionals from the American Heart Association/American Stroke Association. Stroke, 43, 1711–1737. 10.1161/STR.0b013e3182587839 22556195

[brb31517-bib-0007] Diringer, M. N. , Bleck, T. P. , Claude Hemphill, J. I. I. I. , Menon, D. , Shutter, L. , Vespa, P. , … Zipfel, G. . (2011). Critical care management of patients following aneurysmal subarachnoid hemorrhage: Recommendations from the Neurocritical Care Society’s Multidisciplinary Consensus Conference. Neurocritical Care, 15, 211–240. 10.1007/s12028-011-9605-9 21773873

[brb31517-bib-0008] Drake, C. (1988). Report of World Federation of Neurological Surgeons Committee on a universal subarachnoid hemorrhage grading scale. Journal of Neurosurgery, 68, 985–986. 3131498 10.3171/jns.1988.68.6.0985

[brb31517-bib-0009] Duan, W. , Pan, Y. , Wang, C. , Wang, Y. , Zhao, X. , Wang, Y. , & Liu, L. . (2018). Risk factors and clinical impact of delayed cerebral ischemia after aneurysmal subarachnoid hemorrhage: Analysis from the China National Stroke Registry. Neuroepidemiology, 50, 128–136. 10.1159/000487325 29529609

[brb31517-bib-0010] Etminan, N. , & Macdonald, R. L. (2017). Management of aneurysmal subarachnoid hemorrhage. Handbook of Clinical Neurology, 140, 195–228. 28187800 10.1016/B978-0-444-63600-3.00012-X

[brb31517-bib-0011] Foreman, B. (2016). The Pathophysiology of Delayed Cerebral Ischemia. Journal of Clinical Neurophysiology, 33, 174–182. 10.1097/WNP.0000000000000273 27258440

[brb31517-bib-0012] Francoeur, C. L. , & Mayer, S. A. (2016). Management of delayed cerebral ischemia after subarachnoid hemorrhage. Critical Care, 20, 277 10.1186/s13054-016-1447-6 27737684 PMC5064957

[brb31517-bib-0013] Frontera, J. A. , Claassen, J. , Schmidt, J. M. , Wartenberg, K. E. , Temes, R. , Connolly, E. S. Jr , … Mayer, S. A. . (2006). Prediction of symptomatic vasospasm after subarachnoid hemorrhage: The modified fisher scale. Neurosurgery, 59, 21–27. 10.1227/01.NEU.0000218821.34014.1B 16823296

[brb31517-bib-0014] Frontera, J. A. , Fernandez, A. , Schmidt, J. M. , Claassen, J. , Wartenberg, K. E. , Badjatia, N. ,. … Mayer, S. A. (2009). Defining vasospasm after subarachnoid hemorrhage: What is the most clinically relevant definition? Stroke, 40, 1963–1968. 10.1161/STROKEAHA.108.544700 19359629

[brb31517-bib-0015] Geraghty, J. R. , & Testai, F. D. (2017). Delayed cerebral ischemia after subarachnoid hemorrhage: beyond vasospasm and towards a multifactorial pathophysiology. Current Atherosclerosis Reports, 19(12), 50 10.1007/s11883-017-0690-x 29063300

[brb31517-bib-0016] Grimm, J. W. (2015). Aneurysmal subarachnoid hemorrhage: a potentially lethal neurological disease. Journal of Emergency Nursing, 41, 281–284. 10.1016/j.jen.2014.12.018 25661693

[brb31517-bib-0017] Inoue, T. , Ishida, T. , Inoue, T. , Saito, A. , Ezura, M. , Uenohara, H. ,. … Tominaga, T. (2019). Lectin‐Like Oxidized Low‐Density Lipoprotein Receptor‐1 Levels as a Biomarker of Acute Intracerebral Hemorrhage. Journal of Stroke and Cerebrovascular Diseases: the Official Journal of National Stroke Association, 28, 490–494. 10.1016/j.jstrokecerebrovasdis.2018.10.027 30442557

[brb31517-bib-0018] Komotar, R. J. , Schmidt, J. M. , Starke, R. M. , Claassen, J. , Wartenberg, K. E. , Lee, K. ,. … Mayer, S. A. . (2009). Resuscitation and critical care of poorgrade subarachnoid hemorrhage. Neurosurgery, 64, 397–411. 10.1227/01.NEU.0000338946.42939.C7 19240601

[brb31517-bib-0019] Li, D. , & Mehta, J. L. (2000). Upregulation of endothelial receptor for oxidized LDL (LOX‐1) by oxidized LDL and implications in apoptosis of human coronary artery endothelial cells: Evidence from use of antisense LOX‐1 mRNA and chemical inhibitors. Arteriosclerosis, Thrombosis, and Vascular Biology, 20, 1116–1122. 10.1161/01.ATV.20.4.1116 10764682

[brb31517-bib-0020] Ma, F. X. , Zhou, B. , Chen, Z. , Ren, Q. , Lu, S. H. , Sawamura, T. , & Han, Z. C. . (2006). Oxidized low density lipoprotein impairs endothelial progenitor cells by regulation of endothelial nitric oxide synthase. Journal of Lipid Research, 47, 1227–1237. 10.1194/jlr.M500507-JLR200 16522925

[brb31517-bib-0021] Matsuda, N. , Ohkuma, H. , Naraoka, M. , Munakata, A. , Shimamura, N. , & Asano, K. (2014). Role of oxidized LDL and lectin‐like oxidized LDL receptor‐1 in cerebral vasospasm after subarachnoid hemorrhage. Journal of Neurosurgery, 121, 621–630. 10.3171/2014.5.JNS132140 24949677

[brb31517-bib-0022] Mehta, J. L. , Chen, J. , Hermonat, P. L. , Romeo, F. , & Novelli, G. (2006). Lectin‐like, oxidized low‐density lipoprotein receptor‐1 (LOX‐1): A critical player in the development of atherosclerosis and related disorders. Cardiovascular Research, 69, 36–45. 10.1016/j.cardiores.2005.09.006 16324688

[brb31517-bib-0023] Mericle, R. A. , Reig, A. S. , Burry, M. V. , Eskioglu, E. , Firment, C. S. , & Santra, S. (2006). Endovascular surgery for proximal posterior inferior cerebellar artery aneurysms: An analysis of Glasgow Outcome Score by Hunt‐Hess grades. Neurosurgery, 58, 619–625. 10.1227/01.NEU.0000204127.81249.28 16575325

[brb31517-bib-0024] Misaka, T. , Suzuki, S. , Sakamoto, N. , Yamaki, T. , Sugimoto, K. , Kunii, H. , … Takeishi, Y. . (2014). Significance of soluble lectin‐like oxidized LDL receptor‐1 levels in systemic and coronary circulation in acute coronary syndrome. BioMed Research International, 2014, 649185 10.1155/2014/649185 24895597 PMC4033395

[brb31517-bib-0025] Saito, A. , Fujimura, M. , Inoue, T. , Shimizu, H. , & Tominaga, T. (2010). Lectin‐like oxidized low‐density lipoprotein receptor 1 and matrix metalloproteinase expression in ruptured and unruptured multiple dissections of distal middle cerebral artery: Case report. Acta Neurochir (Wien), 152, 1235–1240. 10.1007/s00701-009-0560-6 19936607

[brb31517-bib-0026] Sakurai, K. , & Sawamura, T. (2003). Stress and vascular responses: Endothelial dysfunction via lectin‐like oxidized low‐density lipoprotein receptor‐1: Close relationships with oxidative stress. Journal of Pharmacological Sciences, 91, 182–186. 10.1254/jphs.91.182 12686739

[brb31517-bib-0027] Sawamura, T. , Kume, N. , Aoyama, T. , Moriwaki, H. , Hoshikawa, H. , Aiba, Y. , … Masaki, T. . (1997). An endothelial receptor for oxidized low‐density lipoprotein. Nature, 386, 73–77. 10.1038/386073a0 9052782

[brb31517-bib-0028] Serrone, J. C. , Maekawa, H. , Tjahjadi, M. , & Hernesniemi, J. (2015). Aneurysmal subarachnoid hemorrhage: Pathobiology, current treatment and future directions. Expert Review of Neurotherapeutics, 15, 367–380. 10.1586/14737175.2015.1018892 25719927

[brb31517-bib-0029] Yang, X. , Peng, J. , Pang, J. , Wan, W. , Zhong, C. , Peng, T. , … Jiang, Y. . (2019). The Association Between Serum Macrophage Migration Inhibitory Factor and Delayed Cerebral Ischemia After Aneurysmal Subarachnoid Hemorrhage. Neurotoxicity Research. 10.1007/s12640-019-00072-4 31267487

[brb31517-bib-0030] Yokota, C. , Sawamura, T. , Watanabe, M. , Kokubo, Y. , Fujita, Y. , Kakino, A. , … Minematsu, K. . (2016). High levels of soluble lectin‐like oxidized low‐density lipoprotein receptor‐1 in acute stroke: an age‐ and sex‐matched cross‐sectional study. Journal of Atherosclerosis and Thrombosis, 23, 1222–1226. 10.5551/jat.32466 27025681 PMC5098922

[brb31517-bib-0031] Zhu, Y. , Jiang, H. , Li, Y. , Weng, Y. , Xu, K. , Zhou, L. , … Zhan, R. . (2019). Serum alkaline phosphatase level is associated with angiographic vasospasm, delayed cerebral ischemia‐caused clinical deterioration, and functional outcome after aneurysmal subarachnoid hemorrhage. Neurocritical Care, 31(3), 466–475. 10.1007/s12028-019-00714-7 31016639

